# The Role and Mechanism of Hyperoside against Depression-like Behavior in Mice via the NLRP1 Inflammasome

**DOI:** 10.3390/medicina58121749

**Published:** 2022-11-29

**Authors:** Aoqi Song, Zhenghua Wu, Wenjuan Zhao, Wenqing Shi, Ru Cheng, Jingjing Jiang, Zhuojun Ni, Han Qu, Xijier Qiaolongbatu, Guorong Fan, Yuefen Lou

**Affiliations:** 1 Department of Pharmacy, Shanghai Fourth People’s Hospital, School of Medicine, Tongji University, Shanghai 200434, China; 2 Department of Clinical Pharmacy, Shanghai General Hospital, Shanghai Jiaotong University School of Medicine, Shanghai 200080, China; 3 School of Pharmacy, Shanghai Jiao Tong University, Shanghai 200240, China

**Keywords:** depression, NLRP1 inflammasome, *Hypericum perforatum*, hyperoside, CXCL1/CXCR2, BDNF

## Abstract

Background and Objectives: *Hypericum perforatum* (HP) is widely used for depressive therapy. Nevertheless, the antidepressant effect and potential mechanism of hyperoside (Hyp), the main active component of HP, have not been determined. Materials and Methods: We performed ultra-performance liquid chromatography–quadrupole-time-of-flight–tandem mass spectrometry (UPLC-Q-TOF-MS/MS) technology to analyze the components in HP. Using data mining and network pharmacology methods, combined with Cytoscape v3.7.1 and other software, the active components, drug-disease targets, and key pathways of HP in the treatment of depression were evaluated. Finally, the antidepressant effects of Hyp and the mechanism involved were verified in chronic-stress-induced mice. Results: We identified 12 compounds from HP. Hyp, isoquercetin, and quercetin are the main active components of HP. The Traditional Chinese Medicine Systems Pharmacology Database (TCMSP), the Analysis Platform, DrugBank, and other databases were analyzed using data mining, and the results show that the active components of HP and depression are linked to targets such as TNF-, IL-2, TLR4, and so on. A potential signaling pathway that was most relevant to the antidepressant effects of Hyp is the C-type lectin receptor signaling pathway. Furthermore, the antidepressant effects of Hyp were examined, and it is verified for the first time that Hyp significantly alleviated depressive-like behaviors in chronic-stress-induced mice, which may be mediated by inhibiting the NLRP1 inflammasome through the CXCL1/CXCR2/BDNF signaling pathway. Conclusion: Hyp is one of the main active components of HP, and Hyp has antidepressant effects through the NLRP1 inflammasome, which may be connected with the CXCL1/CXCR2/BDNF signaling pathway.

## 1. Introduction

Depression, as a common mental illness endangering the physical and mental health of the global population, is one of the most serious diseases, affecting 20% of the world’s population, and is an important factor in the rising suicide rate in the 21st century [1]. Depression affects the quality of life of patients and brings a serious burden to patients, their families, and even society as a whole. It is predicted that, by 2030, the leading cause of the global disease burden will be major depressive disorder [2]. Although some progress has been made in the treatment of depression, the chemically synthesized drugs used to treat it have many side effects, such as insomnia at night, daytime sleepiness, weight gain, and recurrence after stopping the drug [3], that make the treatment process impossible for many people with depression. This greatly hinders the curative effect of depression. With the increasing demand for new antidepressant drugs, Chinese herbal medicine has attracted more and more attention due to its effectiveness, safety, fewer side effects, and ability to improve patients’ quality of life [4].

The application of medicinal plants and natural compounds to diseases is a new trend in clinical medical research. *Hypericum perforatum* (HP) is a perennial herb of the genus. HP is in the Garcinia family, known as St. John’s wort in Europe [5]. It has been used in Chinese folk medicine for hemostasis, anti-inflammatory diseases, gynecological diseases, etc. However, the reason for its popularity can be attributed to the effectiveness of this plant in the treatment of mild-to-moderate depression [6]. It is shown that the antidepressant effect of the main components of HP is likely to be attributed to the combined effects of hyperoside (Hyp), hyperforin, hypericin, and several flavonoids. It remains to be determined which one plays the leading role [7].

Hyp is a natural flavonol glycoside found in various plants. Medical research has found that Hyp exhibits various pharmacological effects, including anticancer, anti-inflammatory, antibacterial, antiviral, antidepressant, blood-vessel-protecting, digestive-system-regulating, and organ-protective effects [8,9,10]. Over recent years, the antidepressant, anti-neurodegenerative, and bone-protective effects of Hyp have also attracted people’s attention. Clinically, several drugs containing or made from Hyp have been widely used [11]. Lipopolysaccharide (LPS), the outer membrane component of Gram-negative bacteria, induces the release of inflammatory cytokines [12]. Hyp improves LPS-induced inflammation via inhibiting NOD-like receptor 3 (NLRP3) and Toll-like receptor 4 (TLR4) signaling pathways [13], and the latest research shows that Hyp inhibits the activation of the NLRP3 inflammasome via up-regulating pituitary adenylate cyclase-activating polypeptide (PACAP), thereby effectively reducing the mptp-induced neuroinflammatory response and protecting dopamine (DA) neurons [14]. In addition, Hyp attenuates corticosteroid-induced neurotoxic damage in PC12 cells via decreasing calcium overload and increasing the expression of brain-derived neurotrophic factor (BDNF), thereby exerting antidepressant effects [15]. Hyp also reverses chronic mild stress-induced cognitive impairment by regulating the BDNF signaling pathway [16]. Hyp inhibits LPS-induced microglial inflammation via the p38 and nuclear factor kappa-B (NF-κB) pathways as well as the secretion of various pro-inflammatory cytokines, including tumor necrosis factor-a (TNF-α), nitrogen monoxide (NO), and interleukin-1β (IL-1β) [17]. Hyp also inhibits the activation of the AIM2 inflammasome and the NLRC4 inflammasome [18] as well as the activation of the NLRP1 inflammatory pathway after myocardial infarction [19]. The antidepressant effect of Hyp is clear and safe, with few side effects, but the mechanism of its antidepressant action remains unclear [15]. Combined with network pharmacology, UPLC-Q-TOF/MS, and experimental verification, this study clarified the effect and potential mechanism of Hyp, the main active ingredient of HP, on depression.

## 2. Materials and Methods

### 2.1. Materials and Chemicals

HP (the plant part name is “whole herb”) was provided by Sigma-Aldrich (Shanghai, China) Trading Co., Ltd. (Kume Shunan, Japan) (Serial number: 05295001). Hyp (an analytical standard, HPLC 98%) was supplied by Shanghai Yuanye Biological Products Co., Ltd. (Shanghai, China) (No. B20631). Paroxetine was provided by Aladdin (Shanghai, China) Trading Co., Ltd. (Serial Number: 110429-35-1).

### 2.2. Animals

Sterile Sprague–Dawley (SD) male rats (170–190 g, 6–8 weeks) and C57BL/6 male mice (7–8 weeks, 20–23 g) were bought by Shanghai Slack Laboratory Animal Company. Rats and mice were kept in their respective animal chambers (24 ± 2 °C, 60 ± 5% rh) on a 12-h dark/light cycle. The animal studies in this article were conducted by the Guide for the Care and Use of Laboratory Animals. Rats and mice were acclimated to water and standard laboratory chow for one week before the experiment. All operations, including those involving rats and mice, were conducted in accordance with the Guide for the Relevant Chinese Laws and the Care and Use of Laboratory Animals and were approved by the Shanghai Jiao Tong University Institutional Animal Care and Use Committee (approval number: A2021145; approval date: 23 December 2021).

### 2.3. Drug Administration in SD Rats

We randomly divided SD rats into two groups: (1) the control group. (*n* = 3) and (2) the HP-300 mg/kg group (300 mg/kg/d, *n* = 3) [20]. The SD rats were treated with saline and HP by gavage for three days. The dose per gavage was 1.5 mL each time, twice a day. At the end of the administration, blood samples were collected from SD rats. We used UPLC-QTOF-MS/MS analysis of plasma samples of water extract of HP in SD rats. After the end of the experiment, the rats were euthanized with the method of cervical spine dislocation: the cervical spine of the rats was dislocated by external force so that the spinal cord and brain were disconnected, resulting in the painless death of the experimental rats.

### 2.4. Plasma Collection

We used Eppendorf tubes to collect the blood samples, which were then centrifuged. Then, we transferred the plasma samples to Eppendorf tubes and stored them in a −80 °C freezer for further analysis. After treating 200 L of plasma with 800 L of methanol, the sample was vortexed for three minutes and centrifuged at 14,000 rpm at 4 °C for ten minutes to precipitate proteins. After centrifugation, the supernatant was collected, transferred to another tube, and evaporated to dryness. The samples were then dissolved in 60% methanol (50 L) and centrifuged at 14,000 rpm for ten minutes for later analysis.

### 2.5. UPLC-Q-TOF/MS Analysis

LC-MS raw data were acquired using the Agilent 1290 Q-TOF/MS and Mass Hunter qualitative software. UPLC separation was performed on the samples using an Agilent Poroshell 120 EC-C18 150 mm, 1.9 mm column, on which each sample was analyzed. The mobile phases consisted of (A) 0.1% formic acid in water and (B) acetonitrile, and the following were the optimum UPLC elution conditions: 0–12 min, 10–20% B; 12–18 min, 20–35% B; 18–20 min, 35–10% B, after 1 min. Analyses were conducted at a flow rate of 0.3 mL/min and at a column temperature of 45 °C. 5 μL was the volume of the injection. Agilent 6545 Q-TOF-MS/MS used the Dual Agilent Jet Stream electrospray ionization source (ESI). MS conditions: positive ion mode. gas temperature, 320 °C; fragment voltage, 175 V; sheath gas flow, 8 L/min; and sheath gas temperature, 350 °C, respectively. The range of data acquisition was from 100 to 1000 *m/z*. Data acquisition was automatically calibrated. With the MS/MS mode, argon was used as the collision gas, and the collision energy ranged from 10–40 eV (from low to high).

### 2.6. Network Pharmacology Data Preparation and Network Construction

The Traditional Chinese Medicine Systems Pharmacology Database and Analysis Platform (TCMSP, http://tcmspw.com/tcmsp.php; 1 December 2021) is a unique systematic pharmacology platform for traditional Chinese medicine, highlighting the role of systematic pharmacology in traditional Chinese medicine. Using the “*Hypericum perforatum*” filter as an analysis platform, the TCMSP database was used to retrieve all active ingredients of HP with an oral bioavailability (OB) of 30% and drug-likeness (DL) of 0.18 as screening criteria. The candidate genes were corrected and identified using the *UniProt* (https://sparql.uniprot.org/; 15 December 2021) database. We collected depression-related gene targets in the DrugBank database (http://www.drugbank.ca/; 25 December 2021) using the keywords “major depression”, “depression”, or “unipolar depression”. VENNY2.1 (https://bioinfogp.cnb.csic.es/tools/venny/; 29 December 2021) was used to obtain the Venn diagram, which showed the intersection of depression and HP. The gene ontology (GO) function (cell function, molecular function, and biological function) analysis and Kyoto Encyclopedia of Genes and Genomes (KEGG) pathway enrichment of co-action targets were conducted via the David database (https://david.ncifcrf.gov/; 30 December 2021). The common targets of active components and potential targets of depression were collected and input into the Cytoscape 3.7 software to construct a component–target–disease interaction network that visualized and integrated topological parameters. The STRING database builds functional connections in protein networks by collecting and integrating all known and predicted protein functional associations. The identified potential proteins were imported into STRING (https://string-db.org/; 31 December 2021) to obtain the corresponding relationships of protein interactions, and protein–protein interaction (PPI) networks were constructed by Cytoscape 3.7 software.

### 2.7. Chronic Stress Process

**Figure 1:**
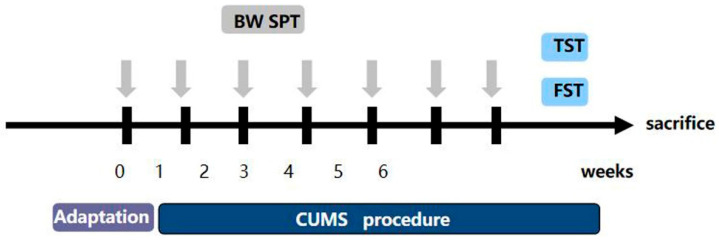
Experimental modeling process.

Research has shown that the most generally reliable, used, and effective depression model is chronic unpredictable mild stress (CUMS) [21]. The CUMS are slightly different from what was previously reported [22]. Mice were isolated in separate cages, and the CUMS procedure consisted of the following stressors: (1) fasting and no water (24 h); (2) forced water swimming at 45 °C (10 min); (3) forced water swimming at 4 °C (10 min); (4) 45° cage tilt (24 h); (5) light/dark reversion (24 h); (6) pinching the tail (5 min); (7) wet cage (24 h); (8) shaking the cage (10 min); (9) tail suspension (10 min); (10) behavior restriction (2 h); (11) an empty cage (24 h). The depression model consisted of eleven stimuli, one of which was randomly selected each day. Each stimulus was administered no more than three times for six consecutive weeks. The flow chart is shown in Figure 1. We randomly divided the mice into the following four groups: (1) the control group (*n* = 5), (2) the CUMS group (*n* = 5), (3) the CUMS+Hyp (36 mg/kg) group (*n* = 5) [19], and (4) the CUMS+Paroxetine (1.8 mg/kg) group (*n* = 5). We gave mice saline, Hyp, or paroxetine by gavage for four weeks. Paroxetine was used as a positive control for antidepressants. After the experiment, the mice were euthanized using the cervical spine dislocation method (the specific method is the same as that used on rats).

### 2.8. Sucrose Preference Test (SPT)

The degree of the animal’s response to the reward is reflected in the consumption of sugar water, and the interest of humans was simulated by the consumption of sucrose water by the mice. The day after the last stress session, in order to measure the anhedonia as previously described, the SPT was performed [23]. Then, all the mice were deprived of water and food for 12 h, and the SPT experiment began in earnest the next morning. Each mouse was given two equal-volume drinking bottles, one containing 1% sucrose and the other pure water. The whole experiment lasted 24 h. To avoid the influence of bottle position, we changed the position of bottles every six hours. At the end of the SPT, we measured the intake of sugar water and pure water for each mouse.

### 2.9. Tail-Suspension Test (TST)

As previously introduced, the tail-suspension test was performed [24]. To begin the formal TST experiment, the mouse’s tail was suspended from a plastic rod with duct tape, and the mouse’s head was down. The distance from the mice’s head to the ground was about 50 cm. During the experiment, the mice’s limbs were not allowed to grasp the tape to avoid affecting the results of the experiment. We suspended all mice for six minutes, and after two minutes, when the mice were passively suspended and remained completely motionless, we recorded the total immobility time.

### 2.10. Forced Swim Test (FST)

To further determine the depression-like behavior of mice, we performed FST experiments. Studies have shown that the forced swim test is a highly reliable test for assessing depressive-like behavioral states in mice [25]. The experimental mice were put into a transparent glass beaker (30 cm in diameter, 50 cm in height) with 30 cm of water in the glass (the water temperature was kept at 25 ± 1 °C). Immobility was recorded as the mice floated in the water in an upright position, not struggling but needing to make some slight movements to keep the head above the water’s surface to avoid drowning. These behaviors indicated the mice were in a state of depression. We used a camera to monitor and record the entire experimental process for six minutes. Each mouse was adapted for three minutes, and the immobility time during the last four minutes was recorded. When the test was over, the mice were removed from the water, instantly towel-dried, and returned to their cages.

### 2.11. RT-PCR

At the end of all the behavioral experiments, we killed the mice and harvested their brains. Total RNA was extracted from mouse hippocampus tissues using the TRIzol reagent (Invitrogen, Waltham, MA, USA) and other chemical reagents. Then, we reverse-transcribe RNA into cDNA using the Prime Script First Strand cDNA Synthesis Kit (Takara Biotechnology, Kusatsu, Japan). We used standard methods to PCR-amplify cDNA. Primer sequences required for this experiment are shown in Table 1. Each group was assigned three replication experiments with β-actin as an internal control. At the elongation stage, we collected the fluorescence signal, calculated the relative levels of mRNA, and analyzed the data using the 2^−ΔΔCT^ method.

### 2.12. Statistical Analysis

All experimental data were analyzed with GraphPad Prism. The data are represented as means± SEM. We used an unpaired, two-tailed Student’s *t*-test or one-way analysis of variance (ANOVA) to compare differences. At *p* < 0.05, statistically significant differences were indicated.

## 3. Results

### 3.1. Screening Hyp from HP by UPLC-Q-TOF/MS

To explore the absorption of HP in vivo, we must know its complex chemical composition. HP extract was qualitatively analyzed using UPLC-Q-TOF/MS. To begin, we created a library of HP ingredients (primarily Hyp). In the literature, we summarized and qualitatively analyzed 12 components of HP. Based on ChemSpider, the name and formula of the compound, the molecular weight, and the chemical structure of the compound were provided. To improve the peak capacity and resolution of HP absorption, we optimized the UPLC-Q-TOF/MS condition.

**Figure 2:**
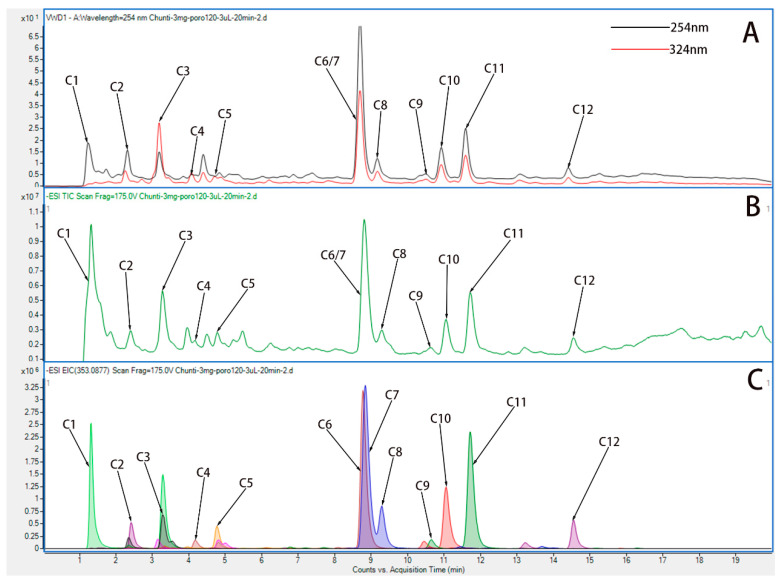
UPLC-Q-TOF chromatograms of water extract of HP. (**A**) Variable wave length ultraviolet detector (VWD) chromatogram of water extract of HP in ESI+ mode. (**B**) Total ion chromatogram (TIC) chromatogram of water extract of HP in ESI+ mode. (**C**) Extracted ion chromatograms (EIC) chromatogram of water extract of HP in ESI+ mode.

By means of comparing all known compounds with reference standards, they were identified. References were from UPLC-Q-TOF/MS literature [26,27], and according to their chromatographic and spectrometric data, the structures of unknown compounds were tentatively characterized. Twelve compounds were detected. A description of the MS/MS data and the cleavage pattern of seven reference compounds is provided, and the ion chromatograms of twelve components of HP identified Hyp from HP. Figure 2 and Table 2 show the results of UPLC-Q-TOF/MS with ESI+mode for the reference standard solution. It was observed that HP contains quasi-molecular ions that can be identified as [M-H]-. Further studies were conducted based on the above results in order to investigate whether Hyp could be absorbed following oral administration.

### 3.2. Network Pharmacology Analysis

**Figure 3:**
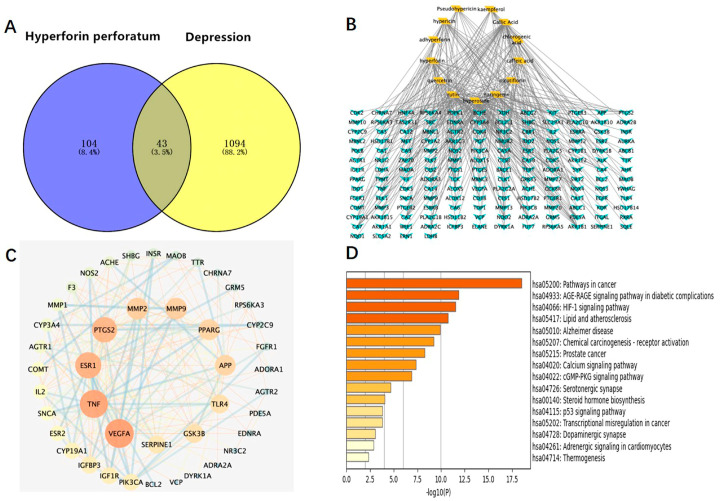
Network pharmacology map of the major active components of HP and depression. (**A**) Venn diagram of related targets of HP and depression. (**B**) The active components of HP and their corresponding anti-depression targets were imported into Cytoscape software to construct the network diagram of the target of the active component. (**C**) PPI network of overlapping targets between drug and disease; the size of the circle represents the target degree. (**D**) KEGG pathway analysis of intersection targets between the main components of HP and depression.

We used the method of VENNY 2.1 software to identify 43 overlapping targets by matching the 104 genes with the genes relevant to disease (Figure 3A). We imported the information about active components and corresponding anti-depression targets of HP into the software of Cytoscape to construct the network diagram of the targets with active components and actions. The same active ingredient can correspond to different targets, which fully indicates that HP anti-depression has the features of multi-targets and multi-components, with a total of 290 intersections and 166 genes. Among them, 22 genes were the intersection targets of Hyp and depression (Figure 3B). The PPI network diagram of intersection targets for HP and depression was made using Metascape. Next, 43 intersection targets of depression and HP were introduced into the STRING online database to build a PPI network model of intersection targets of depression and HP, with 43 nodes and 215 intersections in total. The larger the circle, the more important the target (Figure 3C). As shown in Figure 3D, this includes 16 pathways (*p* < 0.05). The serotonergic synapse, calcium signaling pathway, dopaminergic synapse, and the other 14 signaling pathways relevant to depression were discovered. These pathways were mainly relevant to two pathways of metabolism, four pathways of anti-cancer, three pathways of neuroprotection, two pathways of cardiovascular function, three pathways of signal transduction, and three pathways of anti-inflammation.

### 3.3. Hyp, Isoquercetin, and Quercetin Are the Main Active Components of HP

**Figure 4:**
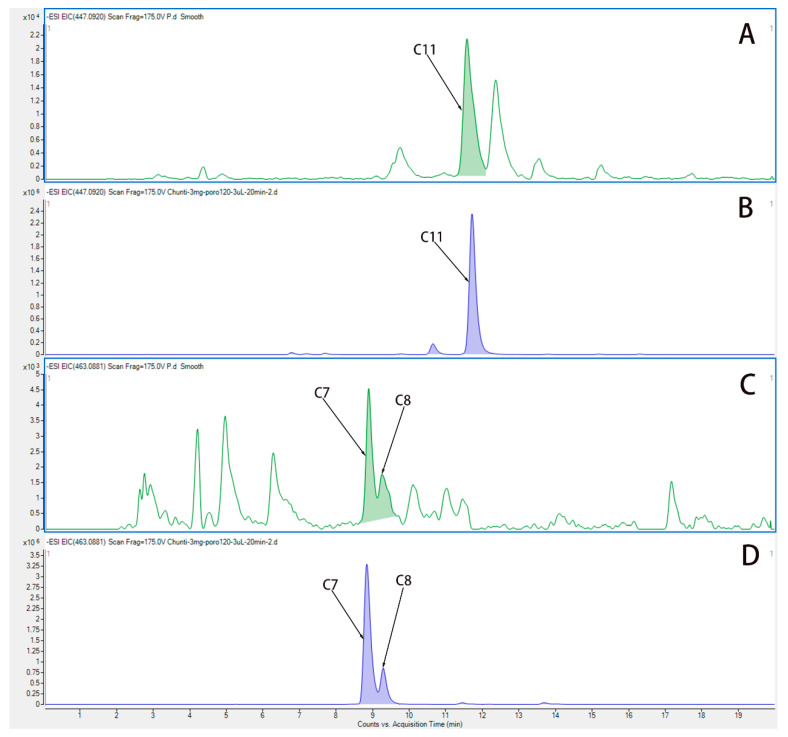
The EIC chromatogram of components in plasma from rats after administration of HP. (**A**) The EIC chromatogram of quercetin (C11) in plasma from rats after the last administration. (**B**) The EIC chromatogram of quercetin (C11) in water extract of HP. (**C**) The EIC chromatogram of Hyp (C7) and isoquercetin (C8) in plasma from rats after the last administration. (**D**) The EIC chromatogram of Hyp (C7) and isoquercetin (C8) in water extract of HP.

We used UPLC-QTOF-MS/MS to analyze the components in plasma from rats after administration of water extract from HP. Figure 4 show the EIC chromatogram of quercetin (C11), Hyp (C7), and isoquercetin (C8) in plasma from rats after the last administration, and the EIC chromatogram of Hyp (C7), isoquercetin (C8), and quercetin (C11) is shown in water extract of HP. Three active components are Hyp, isoquercetin, and quercetin, which were identified in the plasma of rats, and they are the main components in the water extract of HP.

### 3.4. The Intersections of HP Active Component Targets and Depression Targets Were Analyzed by Network Pharmacology

**Figure 5:**
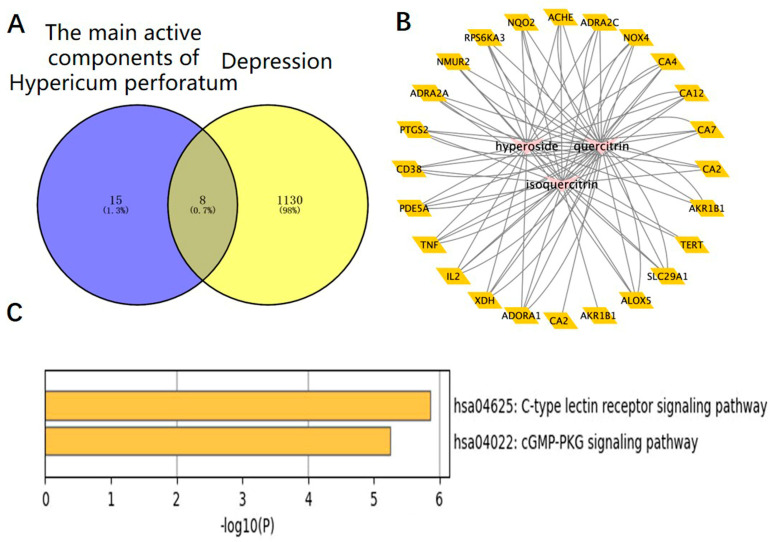
HP’s main active components (Hyp, isoquercetin, and quercetin) and depression: a network pharmacology map. (**A**) Venn diagram of the main active components of HP and depression-related targets. (**B**) The main active components of HP and their corresponding antidepressant targets were imported into Cytoscape software to construct an active component-target network diagram. (**C**) KEGG pathway analysis of intersection targets for Hyp and depression.

The targets corresponding to the main active components of HP were obtained through the SwisStargetPrediction website, and depressive-related genes were obtained through the DisGeNET and OMIM websites. A total of eight intersection targets were obtained after the intersection (Figure 5A). HP’s main active components and depression targets had 28 genes and 876 overlapping genes. Among them, 22 genes were the intersection targets of Hyp and depression (Figure 5B). As shown in Figure 5C, including two pathways (*p* < 0.05), the C-type lectin receptor signaling pathway related to depression was identified. The results are consistent with the anti-inflammatory effects of HP and its main active components [31], its anti-metabolic disease [32], its anti-cancer [33], its neuroprotection [34], its anti-cardiovascular disease [35], and its signal transduction effects [36]. For example, TNF, TLR4, IL-2, BCL-2, etc., are closely related to inflammation, and these targets may be the key targets of Hyp, the main active component of HP, in the treatment of depression. NLRP3, caspase-1, ASC, IL-6, and IL-1β in this signaling pathway are closely related to inflammation and depression. The NLR family, which exists in the central nervous system, consists of the NLRP3 inflammasome and the NLRP1 inflammasome [37]. Studies have shown that the NLRP3 inflammasome mainly exists in microglia, and the NLRP3 inflammasome plays an important role in the occurrence and development of depression [38]. While the NLRP1 inflammasome mainly exists in neurons, studies have shown that it plays an important role in the pathology of neuronal damage and disturbance of consciousness [39] as well as cognitive impairment, a major feature of people with depression. Previous research has shown that the NLRP1 inflammasome-driven inflammatory response in the hippocampus of mice plays an important role in chronic-stress-induced depressive-like behaviors [22,40]. Thus, we surmise that the NLRP1 inflammasome may be a key target of Hyp in depression treatment.

### 3.5. Hyp Ameliorates Depressive-Like Behaviors in Chronic-Stress-Induced Mice

**Figure 6:**
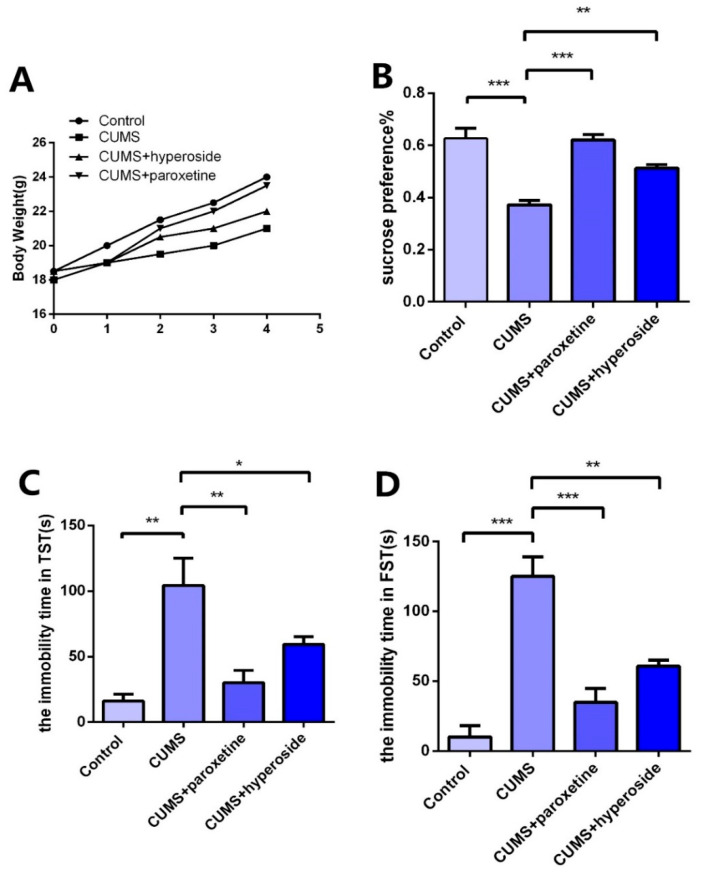
Effects of Hyp on depressive-like behaviors of chronic stress-induced mice. (**A**) Compared with the control group, the CUMS group had a slower rate of body weight growth. Body weight growth was faster in the CUMS+paroxetine and CUMS+Hyp groups than in the CUMS group. (**B**) Hyp and paroxetine significantly increased the sucrose preference rate of CUMS mice in the sucrose preference test. (**C**) Compared with the control group, the CUMS group increased the immobility time of TST. TST immobility time was significantly reduced by CUMS+paroxetine and CUMS+Hyp. (**D**) The immobility time of the FST was increased in the CUMS group compared with the control group. CUMS+paroxetine and CUMS+Hyp significantly decreased the immobility time of FST. Results are shown as the mean from multiple experiments, *n* = 5, one-way ANOVA, followed by Dunnett multiple comparison test; * *p* < 0.05, ** *p* < 0.01, and *** *p* < 0.001.

In order to further explore the antidepressant effect of Hyp, we established a CUMS depression model and administered Hyp for treatment. After the depression model was completed, we tested the depression-like behaviors with the TST, FST, and SPT. Compared to the control group, the CUMS group indicated slow body weight gain (Figure 6A), significantly improved immovability time in TST (Figure 6C) and FST (Figure 6D), and obviously reduced SPT (Figure 6B) in the CUMS group. The results showed that the CUMS mice had obviously depressive-like behaviors, indicating that the depression model was built successfully. Compared to the CUMS group, the immobility time in FST (Figure 6D) and TST (Figure 6C) was obviously reduced in the CUMS+ paroxetine group and CUMS+ Hyp group, and the SPT (Figure 6B) of CUMS treatment mice was significantly increased, indicating that Hyp can improve depressive-like behaviors in chronic-stress-induced mice.

### 3.6. Hyp Decreases NLRP1 Inflammasome Expression in CUMS-Treated Mice

**Figure 7:**
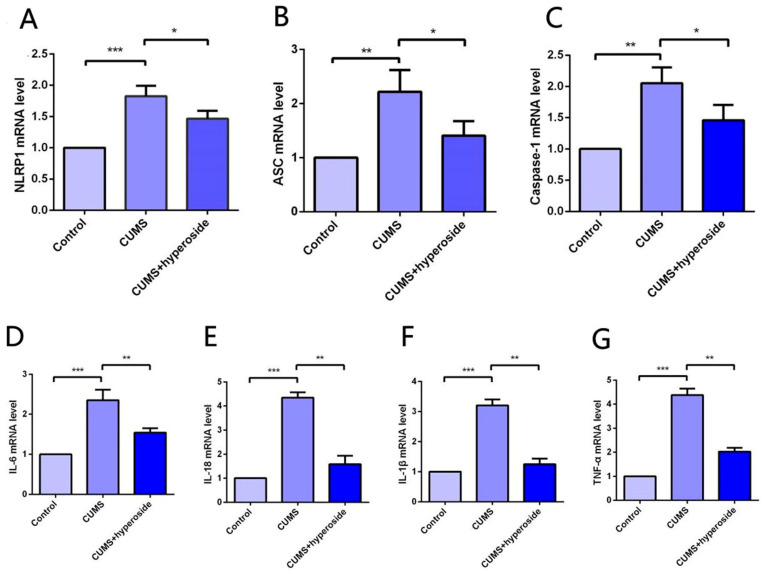
Hyp reduced NLRP1 inflammasome activation and inflammatory response in the hippocampus of chronic-stress-induced mice. (**A**–**C**) Statistical results show that Hyp decreased the mRNA expression of (**A**) NLRP1, (**B**) ASC, and (**C**) caspase-1 in the hippocampus of chronic stress-induced mice. (**D**–**G**) Statistical results show that Hyp reduced the mRNA levels of (**D**) IL-6, (**E**) IL-18, (**F**) IL-1β, and (**G**) TNF-α in the hippocampus of chronic-stress-induced mice. Results are shown as the mean from multiple experiments, *n* = 5, one-way ANOVA, followed by Dunnett multiple comparison test; * *p* < 0.05, ** *p* < 0.01, and *** *p* < 0.001.

The expression of the NLRP1 inflammasome complex in the hippocampus was detected by the method of PCR [22,40]. Our experimental results indicated that Hyp obviously reduced the mRNA levels of NLRP1, ASC, and caspase-1 in the hippocampus of chronic-stress-induced mice (Figure 7A–C). Furthermore, our findings showed that Hyp significantly reduced the levels of inflammatory cytokines such as IL-6, IL-1β, IL-18, and TNF-α in the hippocampus of chronic-stress-induced mice (Figure 7D–G). These results suggest that Hyp can effectively reduce NLRP1 inflammasome expression and its mediated inflammatory response, thereby improving depression-like behavior in mice.

### 3.7. Hyp Downregulated Expression of CXCL1/CXCR2 and Upregulated BDNF e = Expression in CUMS-Treated Mice

**Figure 8:**
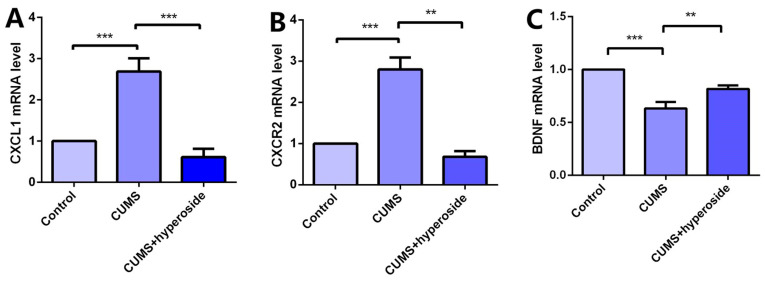
Hyp reduced CXCL1/CXCR2 mRNA levels and increased BDNF mRNA levels in the chronic-stress-induced mice. (**A**) Hyp reduced CXCL1 mRNA level expression in chronic-stress-induced mice. (**B**) Hyp reduced CXCR2 mRNA level expression in chronic-stress-induced mice. (**C**) Hyp prevents the downregulation of BDNF mRNA levels in chronic-stress-induced mice. Results are shown as the mean from multiple experiments, *n* = 5, one-way ANOVA, followed by Dunnett multiple comparison test; ** *p* < 0.01, and *** *p* < 0.001.

Chronic stress induces an inflammatory response, which downregulates BDNF and promotes CXCL1 secretion. Chronic stress activates the expression of the NLRP1 inflammasome, which triggers this inflammatory response [22,40]. To research the influence of Hyp on the depression-like behavior of mice mediated by the NLRP1 inflammasome, we detected the mRNA levels of CXCL1, CXCR2, and BDNF in the hippocampus of mice. Compared with the respective control group, the levels of CXCL1 (Figure 8A) and CXCR2 (Figure 8B) in the hippocampus of the CUMS group were increased, while the level of BDNF (Figure 8C) was decreased. Hyp meaningfully reduced the mRNA levels of CXCL1 and CXCR2 in the CUMS group while reversing the mRNA levels of BDNF. The experimental results show that Hyp down-regulates CXCL1 and CXCR2 and up-regulates BDNF expression in the chronic-stress-induced mice, suggesting that Hyp may inhibit the NLRP1 inflammasome in the chronic-stress-induced mice through the CXCL1/CXCR2/BDNF signaling pathway.

## 4. Discussion

Several clinical trials have conducted relevant studies on the manifestations and characteristics of various mental disorders [41,42,43,44,45]. HP has been used as a medicinal plant for centuries and is regarded as the only herbal alternative to classic synthetic antidepressants in the therapy of mild-to-moderate depression [41,42]. Interestingly, the key constituent responsible for HP’s antidepressant properties, hyperforin, bears little structural or functional resemblance to any known therapeutically used antidepressant [43]. Hyp is one of the main active components in the antidepressant effect of HP [44]. Research has indicated that Hyp has a variety of pharmacological activities, including anti-oxidation, anti-depression, anti-myocardial infarction, anti-inflammatory, and so on [17,45,46]. Presently, tricyclic antidepressants as well as selective serotonin (5-HT) reuptake inhibitors are the most common antidepressants [47]. Despite the high prevalence of depression, depression treatments worldwide fall short of effectiveness and safety. Although some progress has been made in the treatment of depression, chemical and synthetic drugs used for the treatment of depression disrupt the treatment process because of their many side effects and high cost. The discovery of anti-depressant Chinese herbs may bring many benefits to people with depression [48]. Research has indicated that Hyp exerts its antidepressant effects via the ERK-BDNF signaling pathway of extracellular signal-regulated kinase [49]. Hyp was first tested in the FST for its antidepressant activity [50]. Nevertheless, because of the multi-target and multi-ingredient features of traditional Chinese medicine, its pharmacodynamic mechanism is still unclear. Hence, it is imperative that we use the network pharmacology method, such as main component screening, signaling pathway analysis, and drug targets, to explore the possible mechanism of Hyp in the treatment of depression.

In this experiment, UPLC-QTOF-MS/MS was used to detect the water extract of HP, and 12 main compounds, including Hyp, were obtained. They play a role in pharmacological effects by influencing 43 overlapping genes that exert themselves in the treatment of HP. The activity-target network diagram of HP was constructed by introducing the activity-target information into the Cytoscape software. As can be seen from the result, the same active compound can correspond to different targets, which fully indicates that HP anti-depression has the features of multi-targets and multi-ingredients, with a total of 166 genes and 290 intersections. There were 22 Hyp and depression intersection targets among them, and 43 potential HP targets in the treatment of depression were involved 16 related signaling pathways. In addition, the C-type lectin receptor signaling pathway is the most important signaling pathway. In addition, UPLC-QTOF-MS/MS analysis of plasma samples of water extract of HP in SD rats confirmed that the main components in blood were Hyp, isoquercetin, and quercetin. We performed network pharmacology analysis on the intersection of HP’s main active component targets and depression targets. Therefore, these findings prove that Hyp has multi-targets and multi-ingredient synergistic influences and exerts its efficacy, providing a basis for the study of multi-component and multi-target synergistic effects.

BDNF belongs to the family of neurotrophins and plays a critical role in the survival, development, and maintenance of the nervous system [51]. Many studies have shown that BDNF is implicated in the pathophysiology of depression and antidepressant efficacy [52]. Chemokines are small chemotactic cytokines that can induce chemotaxis and migration of immune cells and play an important role in neurogenesis, neuron–glia communication, synaptic transmission, and plasticity [53]. A recent study showed that CXCL1 and its receptor CXCR2 are upregulated in CUMS-induced depressive-like mice. CXCL1 overexpression in the hippocampus induced depressive-like behavior and decreased BDNF levels, whereas CXCR2 inhibition blocked depressive-like behavior and restored BDNF levels [54]. Our previous report demonstrated that the NLRP1 inflammasome mediated chronic stress-induced depressive-like behaviors via controlling CXCL1/CXCR2-BDNF signaling in mice [22,40]. The in vivo experiment confirmed the influence of Hyp in chronic-stress-induced mice. Compared to the control group, the CUMS group indicated slow body weight gain, significantly improved immobility times in the FST and TST, and a significantly reduced sucrose preference test rate. The results showed that the mice had obvious depressive-like behavior, indicating that the depression model had been established successfully. Compared to the CUMS group, the immobility time was reduced in the paroxetine group and the Hyp group from FST and TST, and the sucrose preference test rate of the CUMS group was improved, indicating that Hyp can improve depressive-like behavior in mice. The expression levels of NLRP1, ASC, and caspase-1 in the hippocampus of mice in the CUMS group were higher than in the control group, while they were lower in the CUMS+Hyp group. Furthermore, Hyp reduced the mRNA levels of pro-inflammatory cytokines such as IL-18, IL-6, TNF-α, and IL-1β in the hippocampus of chronic-stress-induced mice. These results suggest that Hyp can effectively reduce NLRP1 inflammasome expression and its mediated inflammatory response, thereby improving depression-like behavior in mice. Compared to the control group, the mRNA levels of CXCL1 and CXCR2 in the hippocampus of chronic stress-induced mice were improved, while the expression levels of BDNF were reduced. The CUMS+Hyp group significantly increased CXCL1 and CXCR2 mRNA levels while decreasing BDNF mRNA levels. Together, these data suggest that Hyp mitigated the role of chronic stress induction in mice. This research showed that Hyp is the main antidepressant component of HP, and its antidepressant effect may be through the NLRP1 signaling pathway to improve depression-like behavior in mice. These effects could be mediated by the CXCL1/CXCR2/BDNF signaling pathway. This research opens up broad prospects for the clinical application of Hyp and has critical theoretical and clinical significance.

## 5. Conclusions

Hyp is the main antidepressant component of HP. Its antidepressant effect may be through the NLRP1 inflammasome. CXCL1/CXCR2/BDNF signaling pathway may mediate the effect. These research findings supply an experimental basis for the clinical application of Hyp in depression treatment.

## Figures and Tables

**Table 1:** The PCR primer sequence.

Gene	Forward Primer, 5′–3′	Reverse Primer, 5′–3′
NLRP1	5-GCTGAATGACCTGGGTGATGGT-3	5-CTTGGTCACTGAGAGATGCCTG-3
ASC	5-CTTGTCAGGGGATGAACTCAAAA-3	5-GCCATACGACTCCAGATAGTAGC-3
Caspase-1	5-TCCGCGGTTGAATCCTTTTC-3	5-CCTTTCCAACAGGGCGTGAA-3
CXCR2	5-CTCTATTCTGCCAGATGCTGTCC-3	5-ACAAGGCTCAGCAGAGTCACCA-3
CXCL1	5-ACTGCACCCAAACCGAAGTC-3	5-TGGGGACACCTTTTAGCATCTT-3
BDNF	5-TCATACTTCGGTTGCATGAAGG-3	5-AGACCTCTCGAACCTGCCC-3
ACTIN	5-TTCCTTCCTGGGTATGGAAT-3	5-GAGGAGCAATGATCTTGATC-3
IL-6	5-CTGCAAGAGACTTCCATCCAG-3	5-AGTGGTATAGACAGGTCTGTTGG-3
IL-18	5-TATCGACCGAACAGCCAACG-3	5-GATAGGGTCACAGCCAGTCC-3
IL-1β	5-GAAATGCCACCTTTTGACAGTG-3	5-TGGATGCTCTCATCAGGACAG-3
TNF-α	5-CCTGTAGCCCACGTCGTAG-3	5-GGGAGTAGACAAGGTACAACCC-3

**Table 2:** Main chemical constituents of water extract of HP [28,29,30].

NO.	Name	Formula	RT/Min	Cal [M-H]-	[M-H]-	Diff (ppm)	Fragment Ions
C1	Quinic acid	C_7_H_12_O_6_	1.18	191.0560	191.0561	0.52	173.0452,127.0396,111.0083
C2	Pyrocatechol	C_6_H_6_O_2_	2.42	109.0294	109.0292	−1.83	90.0111,80.0262,79.0184
C3	Chlorogenic acid	C_16_H_18_O_9_	3.28	353.0878	353.0875	−0.79	191.0562,173.0454,135.0447
C4	Caffeic acid	C_9_H_8_O_4_	4.17	179.0350	179.0349	−0.34	135.0450,134.0372,111.0079
C5	Catechin	C_15_H_14_O_6_	4.77	289.0717	289.0712	−1.87	245.0811,123.0448,125.074
C6	Rutin	C_27_H_30_O_16_	8.78	609.1461	609.1464	0.53	343.0466,300.0278.301.0347
C7	Hyperoside	C_21_H_20_O_12_	8.83	463.0882	463.0883	0.26	300.0279,.0346,271.0247
C8	Isoquercetin	C_21_H_20_O_12_	9.28	463.0882	463.0883	0.26	300.0275,301.0342,271.0239
C9	Kaempferol 3-O-glucoside	C_21_H_20_O_11_	10.65	447.0933	447.0933	0.09	300.0281,301.0350,271.0246
C10	Guaijaverin	C_20_H_18_O_11_	11.05	433.0776	433.0781	1.13	300.0278,301.0348,271.0247
C11	Quercetin	C_21_H_20_O_11_	11.72	447.0933	447.0941	1.88	300.0283,301.0342,271.0247
C12	Protohypericin	C_30_H_18_O_8_	14.72	505.0971	505.0980	1.78	300.0268,301.0345,271.0243

## Data Availability

The data presented in this study are available on request from the corresponding author. The data are not publicly available due to privacy restrictions.
